# A New Predictive Technology for Perinatal Stem Cell Isolation Suited for Cell Therapy Approaches

**DOI:** 10.3390/mi12070782

**Published:** 2021-06-30

**Authors:** Silvia Zia, Giulia Martini, Valeria Pizzuti, Alessia Maggio, Giuliana Simonazzi, Pierluigi Reschiglian, Laura Bonsi, Francesco Alviano, Barbara Roda, Andrea Zattoni

**Affiliations:** 1 Stem Sel srl., 40127 Bologna, Italy; silvia.zia@stemsel.it (S.Z.); pierluigi.reschiglian@unibo.it (P.R.); barbara.roda@unibo.it (B.R.); 2 Unit of Histology, Department of Experimental, Diagnostic and Specialty Medicine, Embryology and Applied Biology, University of Bologna, 40126 Bologna, Italy; giulia.martini18@studio.unibo.it (G.M.); valeria.pizzuti3@unibo.it (V.P.); Alessia.maggio4@studio.unibo.it (A.M.); laura.bonsi@unibo.it (L.B.); francesco.alviano@unibo.it (F.A.); 3 Unit of Nephrology, Department of Experimental, Diagnostic and Specialty Medicine, Dialysis and Renal Transplant, St. Orsola-Malpighi University Hospital, 40138 Bologna, Italy; 4 Obstetric Unit, Department of Medical and Surgical Sciences, Policlinico St. Orsola-Malpighi, University of Bologna, 40126 Bologna, Italy; giuliana.simonazzi@unibo.it; 5 Department of Chemistry G. Ciamician, University of Bologna, 40126 Bologna, Italy

**Keywords:** fetal stem cells, amniotic epithelial cells, isolation protocol, quality control, label-free sorting, diagnostic tool

## Abstract

The use of stem cells for regenerative applications and immunomodulatory effect is increasing. Amniotic epithelial cells (AECs) possess embryonic-like proliferation ability and multipotent differentiation potential. Despite the simple isolation procedure, inter-individual variability and different isolation steps can cause differences in isolation yield and cell proliferation ability, compromising reproducibility observations among centers and further applications. We investigated the use of a new technology as a diagnostic tool for quality control on stem cell isolation. The instrument label-free separates cells based on their physical characteristics and, thanks to a micro-camera, generates a live fractogram, the fingerprint of the sample. Eight amniotic membranes were processed by trypsin enzymatic treatment and immediately analysed. Two types of profile were generated: a monomodal and a bimodal curve. The first one represented the unsuccessful isolation with all recovered cell not attaching to the plate; while for the second type, the isolation process was successful, but we discovered that only cells in the second peak were alive and resulted adherent. We optimized a Quality Control (QC) method to define the success of AEC isolation using the fractogram generated. This predictive outcome is an interesting tool for laboratories and cell banks that isolate and cryopreserve fetal annex stem cells for research and future clinical applications.

## 1. Introduction

Advanced therapy medicinal products (ATMP) are medicines based on genes, cells and tissues to treat human diseases. The somatic-cell therapy consists of cells infusion that replaces tissue functions, cures and prevents diseases. In the last decade, cell therapy approaches, and in particular stem cells (SCs) treatments, are increasing. Adult SCs are widely used to treat malignant diseases like leukemia by hematopoietic stem cells transplantation, and Graft Versus Host Disease (GVHD) by bone marrow mesenchymal stem cells (BM-MSCs) for their immunomodulatory capacity [1,2,3,4,5]. Among stem cell types, perinatal SCs have gained attention because they possess wide differentiation potential and tolerogenic ability [6]. Placenta is a rich source of stem cells: mesenchymal and epithelial cells with staminal characteristics can be derived and it was proven their therapeutic potential in various disease models [7]. Amniotic epithelial cells (AECs) derive from the innermost layer of the amniotic membrane, the one in direct contact with the amniotic fluid. They possess the ability to differentiate toward all three germ layers, they are not tumorigenic and they have immunosuppressive features. AECs are cuboidal epithelial cells firmly adherent to a thick basement membrane [8], expressing high levels of epithelial adhesion molecules, such as EpCAM (CD326) and integrin subunits (CD29 and CD49f), while lacking typical stromal markers expression [9]. AECs also express several pluripotency markers including octamer-binding protein 4 (OCT-4), SRY-related HMG-box gene 2 (SOX-2), and Nanog and showed multilineage differentiation capacity [10]. Perinatal cell populations, including AECs, are also characterized by physiological immunomodulatory and anti-inflammatory properties due to their embryonic origin [11]. Pre-clinical studies have shown AECs’ therapeutic effect in neurological disorders, lung injury, liver injury, diabetes, acute kidney failure, cardiovascular diseases and wound healing among many [12,13,14,15,16,17,18,19,20,21,22]. Moreover, AECs have been proved safe and non-tumorigenic upon transplantation, with no expression of telomerase and limited growing potential in culture [10].

Despite their efficacy and safety, AECs expressed differences in stemness characteristics, differentiation potential and immunomodulatory activities depending on the heterogeneity of primary derived cells which lead to a variable effect based on population composition [23,24]. Thanks to their early origin and easy access, AECs could be isolated, cryopreserved in specialized cell banks for future autologous therapy approaches [25]. Despite AECs isolation method is easy and does not require expensive material, the yield of AECs can be quite variable and their characteristics appear to be dependent on the genotype, age of the donor, region of cell isolation on placenta [24], cross-contamination of amniotic epithelial and mesenchymal stromal cell, isolation protocol, epithelial-to-mesenchymal transition of AECs [26] and measuring methods that are used for characterization [27]. Few studies have focused attention on membrane microscopic observation before enzymatic treatment [28] but it is operator-dependent. Several protocols have been proposed for isolation of hAECs with a wide range of yielded cells, viability and purity [29,30].

Reproducible and accurate systems are needed to standardize the isolation protocol of primary SCs. Identification and selection procedures able to isolate stem cells are essential to most cell therapy models. Multiple methods have been developed including mechanical sorting, surface receptors or biological markers of stemness. All these processes involve the knowledge of specific marker expression, which is not always known, and cell manipulation that must be scalable and amenable to GMP procedures. These requirements may be no trivial. Therefore, development of new technologies and relevant application methods are welcomed.

Microfluidic systems are widely used to test quality of pharmaceutical compounds but the working dimensional range belongs to micro-nanoparticles [31]. In the last decade, field-flow fractionation (FFF) has proven its capacity to analyze, discriminate and separate a wide size range of cells mixture based on their physical characteristics with high resolution and throughput [32,33]. In order to work with cells that have the ability to adhere to plastic, a novel method has been developed, the Non-Equilibrium Earth Gravity Assisted Dynamic fractionation (NEEGA-DF) [34]. Adhesion and contact of cells with the fractionation device are totally avoided by in-flow injection, by the absence of stop-flow cell sedimentation, and by using elution flow rate values able to generate hydrodynamic forces that are intense enough to lift and keep cells away from the channel wall. Cells having different physical characteristics acquire different velocity inside the capillary channel and elute at different time, so it is possible to collect the subpopulations composing the biological sample. It was proven that mesenchymal and epithelial cells from different organs origin, showed specific profile outcome of the separation process meaning that this method is suitable to underlined intra-differences in the cell population that other techniques do not do [34]. We developed an automated instrument that implements the NEEGA-DF method (Celector^®^, Stem Sel srl, Bologna, Italy), using a micro-camera for cell detection and a specifically designed software for image acquisition, post-processing and data analysis. The output of the instrument is a multiparametric fractogram representing number, size and shape of the eluted cells as a function of fractionation time and it is the fingerprint of the cell sample.

In this study, we used the instrument Celector^®^ to perform the quality check of freshly isolated AECs, to compare possible differences in cells’ yield and composition of amniotic membrane treated with two concentrations of trypsin, 0.1% and 0.25%. The live fractogram was used as predictive data to define successful isolation procedure and additionally, post-processing image data were compared to biological data of cell recovery, cell vitality and adhesion ability.

## 2. Materials and Methods

### 2.1. Instrumentation

#### 2.1.1. Fractionation Principle and Procedure

The separation is obtained in a capillary device (channel) with rectangular cross section where cells suspensions are eluted through a laminar flow of physiological buffer. When a cell suspension is injected at a flow rate of 1mL min into the system, cells are transported by the flow and reach a specific position across the channel thickness during transportation due to the combined action of gravity, acting perpendicularly to the flow, and opposing lift forces that depend on the morphological features of the sample. Cells at a specific position in the channel acquire well-defined velocities and are therefore eluted at specific times [35]. The in-flow injection, the absence of a stop-flow, cell sedimentation step, and the use of elution flow rate values able to generate hydrodynamic forces that are intense enough to lift and keep cells away from the channel wall, make cells avoid any contact with the device with a consequent maintenance of native properties and high sample recovery.

The fractionation procedure involves at first the decontamination of the fractionation system by flushing with cleaning solution at 1 mL min flow rate. Next, the system was washed copiously with sterile, demineralized water at the same flow rate. Although the NEEGA-DF method is optimized to prevent contact between cells and fractionation device, to block non-specific interaction sites on the plastic walls, the fractionation system was flushed at 0.5 mL min with a sterile coating solution. Finally, it was filled with a sterile mobile phase. All solutions were provided by Stem Sel srl. The instrument is placed under a laminar flow hood to maintain the sterility of the collected cells.

#### 2.1.2. Optical Analysis

Eluted cells were monitored using a micro-camera detector (MER-U3 camera, DAHENG IMAGING, Beijing, China) that is placed at the outlet of the fractionation channel. The imaging software (Celector Optics, Stem Sel srl, Italy) generates real-time fractogram representing the percentage of frame area covered by the cells versus recorded time. The imaging data are then post processed to obtain number and geometrical features of eluted cells as a function of time. In this work, we focused on the area/diameter and circularity of cells to obtain information on population heterogeneity and composition of possible sub-populations. These geometrical features were then visualized as scatter plot and curves using dedicated data processing (Stem Sel Analyzer), to obtain the average of all parameters in a selected time interval (cell fraction).

### 2.2. Cell Analysis and Collection

For every sample, cells were first analyzed to obtain a patient-specific fractogram and identify the fractions to collect. Consecutive analyses were run to increase the number of collected cells per fraction. The fractionated cells were centrifuged, and cell recovery was calculated by erythrosine dye (Sigma, St. Louis, MO, USA) to count alive and dead cells for both groups, 0.1 and 0.25% trypsin.

### 2.3. Isolation of Human Amniotic Epithelial Cells (AECs)

The study was approved by the Local Research Ethics Committee (EM894-2020_68/2017/U/Tess/AOUBo) and written informed consent was obtained from each healthy donor before specimen collection.

Amnion membranes were retrieved from term pregnancies (37–40 weeks of gestation) delivered by Caesarean section. hAECs were isolated using a modified procedure of the protocol previously reported [36], the amnion layer was mechanically peeled off the chorion layer and immediately washed in Phosphate Buffer Saline (PBS, Gibco, ThermoFisher Scientific, Waltham, MA, USA) without calcium and magnesium (HBSS, PAA Laboratories GmbH, Pasching, Austria) until blood clots were completely removed. For each sample, the amnion was minced into 4 to 6 medium size pieces (25 cm^2^ approximately) and divided into two groups: one group was treated with 0.1% of trypsin-EDTA and the other group with a 0.25% trypsin-EDTA for all digestion steps (Gibco). Before enzymatic treatment, membrane was washed in PBS and 0.5 µM EDTA for 10 min then firstly, pieces were incubated for 10 min at 37 °C to exclude debris and then incubated for a second and third 40 min enzymatic digestion using the two concentrations to release the amniotic epithelial cells (AECs). Single cell suspension was washed with PBS and tested for viability with erythrosine dye (Biochrom AG, Berlin, Germany) and the number of viable and dead cells were counted. Cellular pellets were resuspended in the growth medium consisted of DMEM high glucose supplemented with 10% FBS, EGF (10 ng/mL; Sigma-Aldrich, St. Louis, MO, USA) and 1% penicillin and streptomycin (all solutions from Gibco) and plated at a density of 100,000 cells/cm^2^ in a T25 flask for expansion. The leftover cells, at least 1.2 × 10^6^ cells, were analysed using Celector^®^ for quality control of the isolation process and cell sorting.

For the study AECs from the second digestion were diluted to a final concentration of 3 × 10^6^ cells per mL and 100 µL were injected. Cells were automatically re-dispersed 3 times to homogenize the suspension and eluted at a flow rate of 1 mL min.

### 2.4. Downstream Analysis

Collected cells for each fraction and from the control group were seeded at a cell density of 100,000 cells/cm^2^ to observe cell adhesion ability and morphology. In order to define the success of the isolation protocol, cells must adhere to plastic surface and show proliferative ability one week after isolation occur. If cells did not adhere or showed no ability in proliferation, isolation was defined as unsuccessful.

10,000 freshly sorted cells of each group, 0.1 and 0.25% trypsin, were seeded on a glass coverslip. After 4 days, cells were fixed in 10% formalin and stained for nuclear DAPI (Prolonged antifade, Molecular Probes). Images were taken using a fluorescent microscope and analyzed using the NII plugin to determine the number of normal, senescent and mitotic nuclei [37].

### 2.5. Statistical Analysis

Statistical analysis was performed using Graph Pad Prism v 8, running the *t*-test and mean and standard deviation were graphed.

## 3. Results

### 3.1. Predictivity

**Figure 1:**
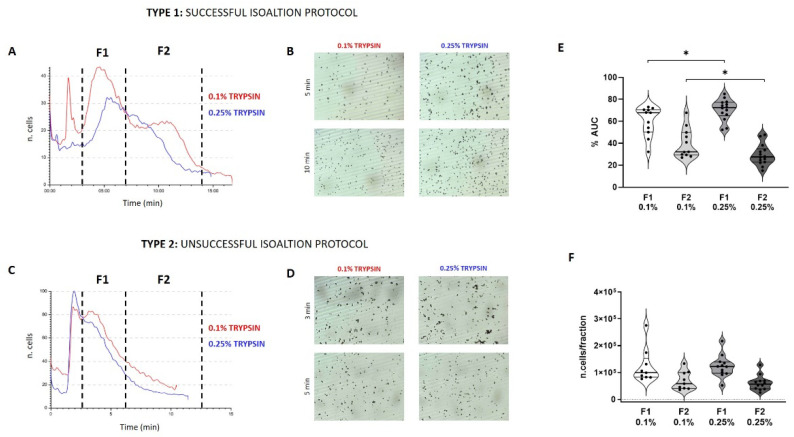
Representative images of a successful isolation protocol of AECs (Type 1) and an unsuccessful (Type 2) protocol. Profile represents the number of cells versus time of analysis (**A**,**C**) for both trypsin treatment 0.1 and 0.25%. The time interval of cells collection is shown as a dotted line that divides the two subpopulations F1 and F2 (F1 from the 3rd to the 7th minute and F2 from the 7th to the 14th minute of analysis). Live images of eluting cells are shown for both groups for type 1 and 2 (**B**,**D**). Cell distribution between F1 and F2 based on the calculation of the area under the curve (AUC) expressed as a percentage compared to the total area of the profile. The difference was seen between the F1 and the F2 of the 0.1 and 0.25% groups (**E**); distribution was also expressed as a number of counted cells by the software for each fraction of all samples analyzed (**F**). (*t*-test: *p* < 0.05 *).

Amniotic epithelial cells (AECs) were isolated using different concentration of trypsin, 0.1 and 0.25%. Freshly isolated cells from the second digestion were immediately analysed by Celector^®^ to profile populations. Two types of profiles were generated: a profile having two distinct peaks (type 1) (Figure 1A) and a profile with all cells eluted in the first minute of the analysis (type 2) (Figure 1C). Type 1 profile represents alive and proliferating cells while type 2 showed unsuccessful protocol, with no cells attaching to the plate and not able to proliferate. For both type of profile, unretained cells and debris eluted in the first minute of the analysis, then first subpopulation eluted between the 3rd and the 7th minute of analysis (Fraction 1, F1), and when present the second sub-population eluted between the 7th and the 14th minute (Fraction 2, F2). No difference was observed between the use of different trypsin concentration; both concentrations showed the same predictivity about the isolation protocol. Cell aggregates were observed in F1 for both trypsin treatments, while single cells eluted in F2 (Figure 1B). Sample belonging to type 2-profile, the one resulting unsuccessful in the cell expansion, presented more and bigger cell aggregates in F1 compared to type 1 samples (Figure 1D) and very few cells eluted in F2.

For both treatments, most of the cells eluted in F1, showing a higher intensity of the peak confirmed by the area under the curve (AUC) (Figure 1E) which represents the number of eluted cells. The distribution of cells between F1 and F2 in the sample treated with a 0.25% trypsin was statistically different compared to 0.1% samples: 0.25% samples generated a higher AUC in F1 and a lower AUC for F2 compared to 0.1% samples. Post-processing analysis of counted cells confirmed the difference between F1 and F2 from both groups, even though it was no significant (Figure 1F). AECs profiling showed the ability of this technology to predict the achievement of the isolation process.

### 3.2. Quality Control (QC) of Freshly Isolated AECs

**Figure 2:**
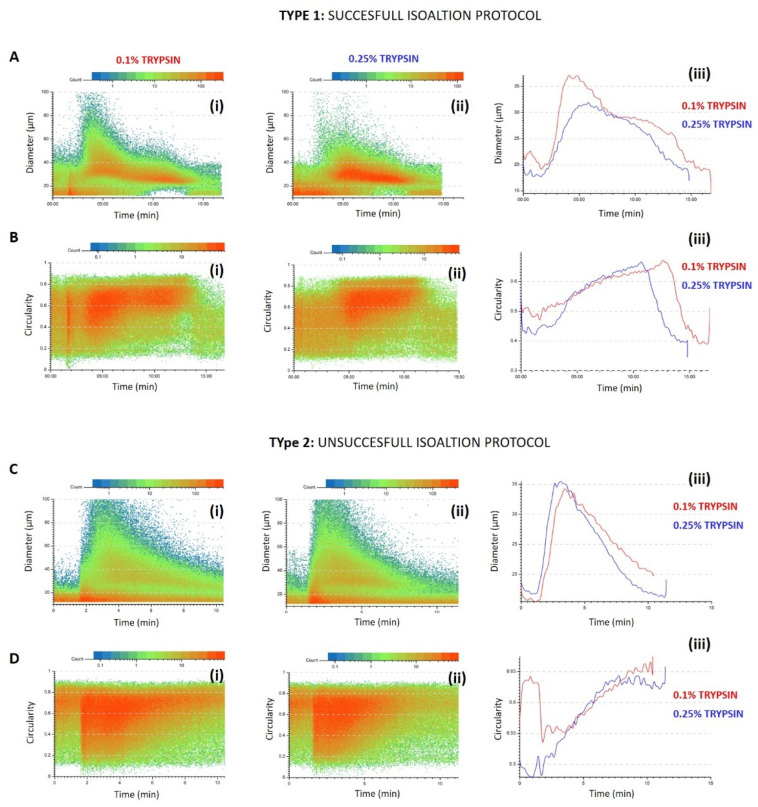
Representative images of the physical parameters of AECs from 0.1 and 0.25% trypsin treatments. Diameter and circularity were analyzed and compared between the two treatments. Scatter plot of diameter (**A**) and circularity (**B**) for 0.1% (**i**), 0.25% (**ii**) and the overly of the average (**iii**) for the successful protocol and for an example of an unsuccessful protocol for the cell diameter (**C**) and circularity (**D**).

The fractionation profile gave the immediate predictivity of the AECs isolation process while the post processing analysis of the recorded data allowed a better characterization of the examined populations. The software presents the physical characteristics of each cell eluted under the camera, becoming in this way an excellent tool to increase the information besides the fingerprint obtained by the profile. Thanks to this tool, we discovered a difference between the 0.1% and 0.25% samples. When the isolation protocol was successful, AECs derived by the 0.1% showed a more heterogenous population, with two clear sub-populations F1 and F2. Scatter plot representing the cell diameter versus the time of analysis showed the two-populations distribution, F1 showed a wider dimensional distribution composed of bigger cells and cell-aggregates and a second population dimensionally more homogenous and smaller in the F2 (Figure 2A(i)). On the contrary, AECs isolated using 0.25% trypsin showed a more homogeneous population in respect of the cell dimension, with a more compact cell cloud around the diameter of 30 µm especially in F1 (Figure 2A(ii)). When cell circularity was studied, the F1 sub-population express the same average in both trypsin treatments while F2 cells of the 0.25% showed the most circular cells at the 10th minute of analysis and the 0.1% at the 13th minute, an indication of how the two concentrations differ in the membrane treatment and consequent cells release. Compared to the 0.1% AECs, 0.25% AECs are a more homogenous population. Analysis of samples that did not retrieve proliferating cells, clearly showed in the scatter plot distribution that all injected cells eluted in F1 and no cells are present at the 10th minute of the analysis which is the highest point of the second peak in type 1 profile.

**Figure 3:**
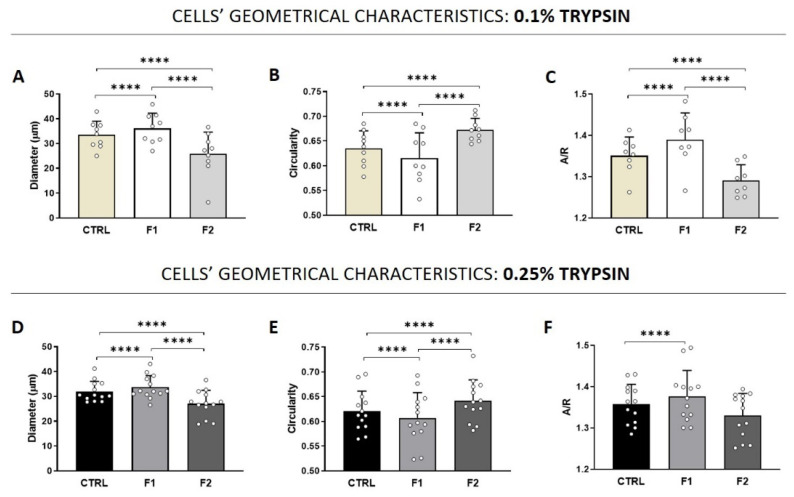
Cells’ geometrical features for AECs derived using 0.1% trypsin (**A**–**C**) and 0.25% trypsin (**D**–**F**) for diameter, circularity and aspect/ratio (A/R). (*t*-test: *p* < 0.0001 ****).

Besides the scatter plot, the average of every parameter was calculated for each fraction and compared to the general population (CTRL). The results confirmed the principles of the separation process because cells with higher diameter are in F1 and smaller one elutes later in F2 (Figure 3A,D). The same trend was observed for the circularity parameter: the most circular cells are in the F2. Stem cells have the characteristics to be circular and with well-defined counters, so there is a link between this parameter and then the ability of adhesion and proliferation of these cells. Even though there is only a small difference, F2-AECs from 0.25% trypsin treatment is slightly more circular than 0.1% AECs. One of the hypotheses is that this treatment released smaller and more circular cells compared to the 0.1% treatment. When cellular aspect/ratio was measured, F2 cells showed a lower value compared to F1 cells, which is in line with the higher circularity of these cells. Despite a significant difference between F1 and F2, the difference between F1 and F2 for 0.25% samples is less highlighted than in 0.1% samples. This result is in line with the ability to isolate a more homogenous population using the 0.25% concentration.

### 3.3. Viability and Cell Recovery

**Figure 4:**
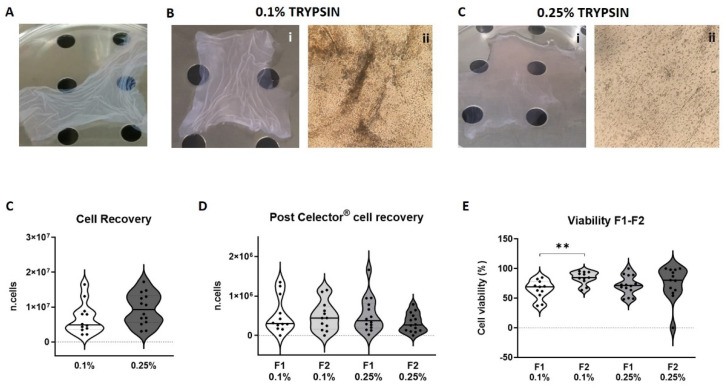
Representative images of amniotic membrane pre-treatment (CTRL) (**A**), macroscopic (**B**-**i**) and microscopic image using a 4x objective (**B**-**ii**) post-treatment using 0.1% trypsin and macroscopic (**C**-**i**) and microscopic image using a 4x objective (**C**-**ii**) post-treatment using 0.25% trypsin; the number of cells recovered after enzymatic treatment counted using the erythrosine solution to discriminate alive cells (**C**); the number of collected cells per analysis (run) for each fraction for both trypsin treatment, 0.1 and 0.25% (**D**); Percentage of viable cells for collected fractions F1 and F2 for both enzymatic treatment (**E**). (*t*-test: *p* < 0.01 **).

Membranes treated with the two concentrations of trypsin gave very heterogenous result on cell recovery. For both the concentrations the distribution is rather wide as shown in the graph (Figure 4C). Even though there was not a significant difference, the membrane treated with a 0.1% trypsin recovered almost half of the cells of 0.25% (4.35 × 10^6^ vs. 8 × 10^6^ cells). At the macroscopical and microscopical observation, membrane treated with 0.1% trypsin resulted whiter and cells were still present on the membrane (Figure 4B(i,ii)) while membrane treated with the 0.25% concentration showed gel-like consistence and very few cells were seen on the membrane (Figure 4C(i,ii)). The 0.25% treatment results more efficient in cell removal as seen by a higher cell recovery (Figure 4D). For the 0.1% samples, cell recovery was equally distributed between F1 and F2 while for the 0.25% treated samples, F2 was less abundant than F1 (Figure 4E). To see the effect of the digestion process, the viability of post-fractionation cell collection was measured by counting the number of alive cells compared to the total cells in each fraction. Statistical difference was seen between F1 and F2 for the 0.1% group with the F1 have been the most vital one, while for the 0.25% group no difference was observed because of the heterogeneity of the F2 group.

### 3.4. Morphology and Adhesion Properties

**Figure 5:**
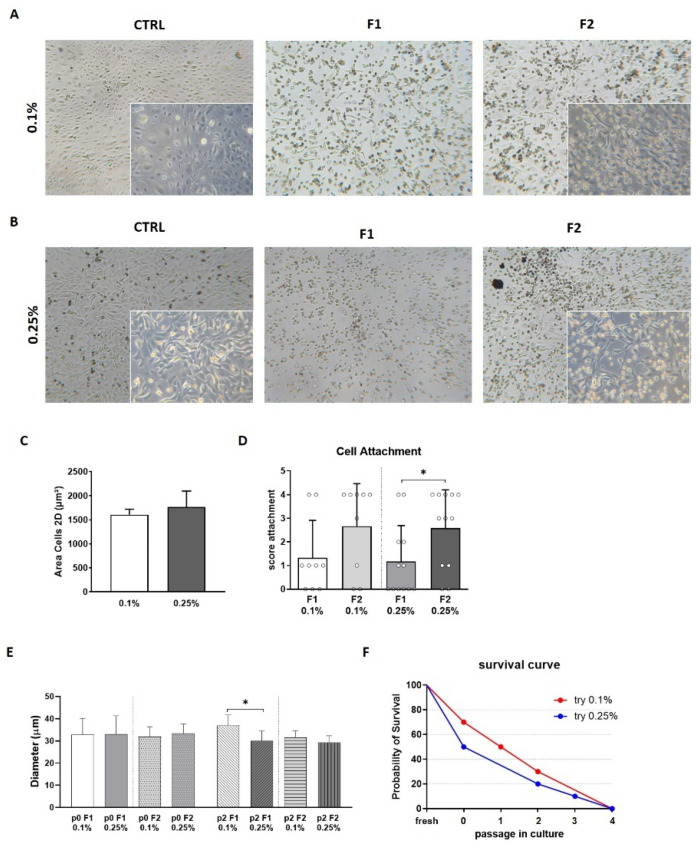
Representative images of AECs from both enzymatic treatment, 0.1% (**A**) and 0.25% (**B**) from control population (CTRL), F1 and F2 derived AECS and it is visible that a minority of the cells in F1 attached to the plate. AECs from 0.1% and 0.25% treatment did not show difference in area size (**C**). The ability to adhere to the plastic surface was scored (from 0 to 4, min to max) for the separated AECs from F1 and F2 for both trypsin groups. F1 derived AECs have a lower ability to adhere compared to the F2 cells, which are the ones more vital and able later to proliferate (**D**). Cell diameter was measured by post-processing analysis and cell population was divided following the same time interval used for the fresh sample. AECs at passage 0 and 2 were analyzed for both trypsin treatments (**E**). AECs control from 0.1 and 0.25% were grown in culture to monitor proliferation ability (**F**). The 0.1% AECs have a higher adhesion propensity (70%) while AECs from 0.25% adhere to the culture dish only in 50% of the cases. Even with the difference in initial adhesion, AECs grow until the 4th passage in culture. (*t*-test: *p* < 0.05 *).

Morphologically, cells from both conditions had an epithelial morphology, small in size and few cells showing long pedicles (Figure 5A). In the 0.1% condition we observed few cells with wider cytoplasm compared to 0.25% which resulted homogenous and there was no difference when adherent cell area was measured (Figure 5C).

AECs from both trypsin treatment, 0.1% and 0.25%, had the ability to adhere to plastic with a different grade, 70% for the 0.1% treated samples and 50% for the 0.25% samples. This difference could be explained by the different grade of adhesion of single fractions. For the 0.1% samples, F2 was mostly adherent and many cells from the F1, whereas in the 0.25% group there was a significant difference between F2 and F1 cells, with the latter showing a lower adhesion profile (Figure 5D). When 0.1 and 0.25% AECs were kept in culture, we saw a similar trend, meaning that the F2 component from 0.25% is probably contributing to the proliferation (Figure 5E).

**Figure 6:**
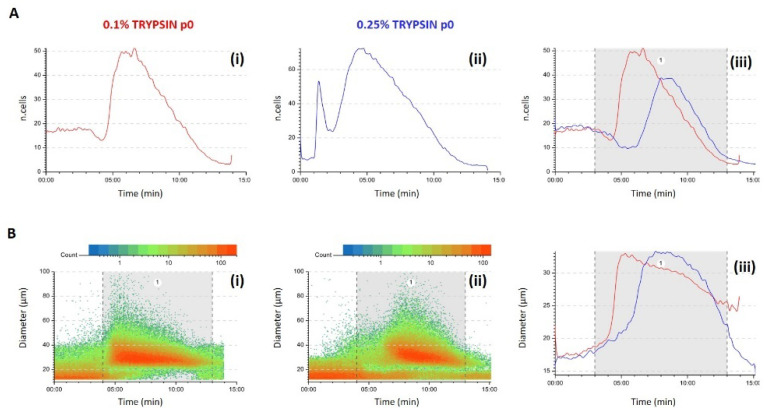
Representative images of the physical parameters of AECs from 0.1% and 0.25% trypsin treatments at passage 0 in culture. Profile was analyzed and compared between the two treatments (**A**). Scatter plot of diameter (**B**) for 0.1% (**i**), 0.25% (**ii**) and the overly of the average (**iii**).

**Figure 7:**
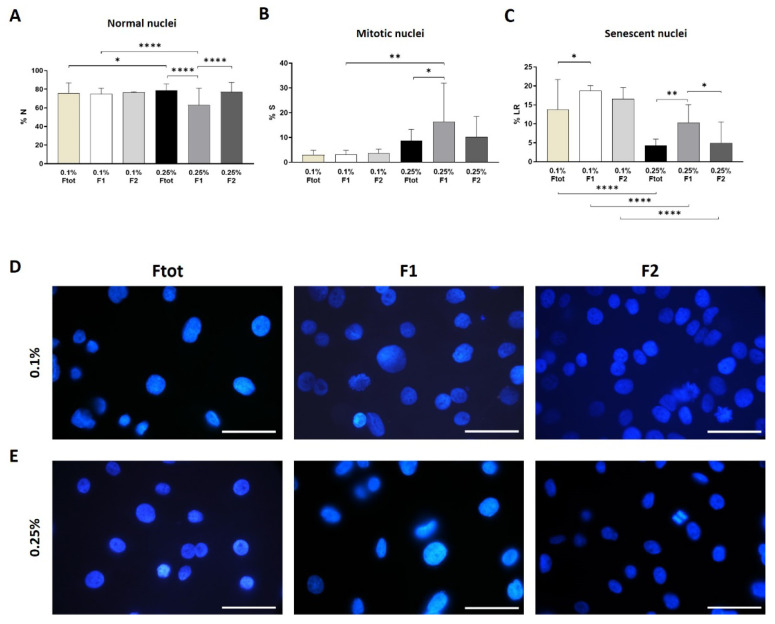
Morphological analysis of the nuclei showed presence of normal nuclei (**A**), mitotic (**B**) and senescent (**C**). Representative images of immunofluorescence staining for DAPI to visualize shape heterogeneity in fractions samples for both enzymatic treatments (**D**,**E**). (*t*-test. *p* < 0.05 *, *p* < 0.01 **, *p* < 0.0001 ****).

Morphologically AECs from both groups have similar adhesion area and could proliferate till the 4th passage in culture. Despite the AECs in adhesion did not show any difference in the 2D cell dimension between the two concentration treatments, scatterplot graphs showed the heterogeneity of the two populations (Figure 6). AECs from 0.1% treatment showed a temporarily wider profile, with cells eluted already from the 4th to the 13th minute while 0.25% AECs eluted from the 7th till the 13th minute. The higher concentration of trypsin performs a selection on the population. To investigate even more the heterogeneity of these cells we investigated the shape and dimension of the nuclei (Figure 6). Imaging analysis of DAPI stained nuclei showed a similar distribution of normal nuclei in the 0.1% samples, with no difference between the F1 and F2 (Figure 7A). Interestingly, we observed a lower presence of normal nuclei in the F1 from 0.25% samples compared to F2 (63 vs. 77%, *p* < 0.0001). Cells derived by 0.1% trypsin treatment had a higher presence of cells with senescent nuclei (LR) compared to 0.25% cells and the majority resides in the F1 both in the 0.25 and 0.1% group (Figure 7C). Cell division was observed in culture and the 0.25% samples seemed to be more proliferating than the 0.1% even though the fraction with the higher presence of mitotic nuclei was the F1.

## 4. Discussion

This study evidenced the potential of this technology as a quality control system to predict the outcome of stem cell isolation procedure and the quality of freshly isolated cells, specifically amniotic epithelial cells (AECs).

Stem cells act following different mechanisms to treat damages: they differentiate into specialized cells, modulate host immuno-response and release specific factors that stimulate tissue regeneration. After 2018, the number of clinical trials using MSCs is slowing down because comparison among trials is difficult due to cells heterogeneity, organ origin, different cell preparation protocols and different passage numbers. Standardization of cells is necessary to obtain more reproducible and comparable results among centers [38,39,40]. In the last decade a huge effort has been made to ameliorate standards for cell culture and production, using optimal reagents, industrialized bioreactors where all variables such as temperature and gas exchange are constantly controlled, in order to avoid operator’s variability. Despite standardize protocols and reagents may help the reproducibility of isolation protocol and the in vitro cell expansion, the inter-patient differences are still under debate. As an example, patient derived induced pluripotent stem (iPS) cells are key platform to study the impact of human cell type-specific gene regulation but inevitably, differences between donors affect most iPSC traits, from cell morphology to DNA methylation, mRNA and protein abundance to pluripotency and differentiation [41]. Even after controlling for genotype, substantial experimental heterogeneity remains.

The therapeutic potential of AECs is well known and recently, it was proven to enhances the engraftment, viability and graft function of pancreatic islet organoids in diabetes model when co-cultured, which can be used as a cell therapy approach for diabetes [19]. The possibility to use these cells in an allogenic manner raised their interest even more in the scientific community [42]. The isolation procedure is very simple consisting in the use of trypsin to detach the epithelial layer from the membrane, leaving the mesenchymal component attached to it. In our records, AECs recovery and proliferative ability were very heterogeneous and could not be linked to a macroscopical observation of the quality of the membrane, therefore we investigate the potential of the NEEGA-DF method as a quality control system. Technically, enzymatic digestion could lead to the detachment of cells in an aggregate form that unlikely adhere to the plate and could hinder single cell attachment. Clearly, cell aggregates are heavier than single cells, so through the method could be separated from the single cells. Moreover, dead and damaged cells are usually eluted in the first minute of the analysis because they do not reach the equilibrium, inferring with the quality of the cells’ suspension.

Results showed the prediction of the live fractogram to define the success of the isolation protocol: bimodal profile is linked to alive, adherent and proliferating cells, whereas fractogram having one single peak or with a low intensity second peak represents the non- or poorly- adherent cells. Cells isolated using both concentrations of trypsin, 0.1 and 0.25%, showed an equal prediction of the fractogram, which means that the general action of the enzyme on the membrane is very similar. This prognostic tool can save time, reagents and effort in culturing only proliferating cells as the verification of vitality required few days because any additional movement of the dish, to observe cell adhesion at the microscope, could affect cell attachment. The QC method developed delivered the answer to the closed-question on the isolation procedure in 15-min time. Count of alive cells is not a very reliable method to define the quality of digestion, since some samples, characterized by a majority of alive cells, showed a single-peak profile and resulted in non-adherent cells. These cells are probably senescent cells unable to adhere to plastic surfaces or damaged cells by the enzymatic treatment, with a consequent selection of the solely proliferative clones. Nuclei analysis showed the presence of senescent cells mostly in F1, both in 0.1% and 0.25% samples, even though the lower concentration treatment showed a higher number of senescent nuclei both in F1 and F2 compared to 0.25% cells. We hypothesize that 0.25% concentration performs a selection, removing senescent cells, and leaving a more homogenous sample as proved by scatter plot graph representing cells’ diameter after first expansion in culture. These cells elute at the same time interval of F2 proving their initial belonging of the F2 peak and the consequent no attachment of mostly F1 cells. Even at later passage, 0.25% AECs showed a more homogeneous dimensional distribution, whereas 0.1%, especially at passage 2 in culture, showed an increase in the area of cells eluted in the F1 interval. We conclude that the 0.25% treatment applied a selection on the most proliferate clones of AECs, resulting in adherent proliferating cells.

We presented for the first time an analytical platform that analyze freshly primary cells and understand the effect of isolation process on their viability and propensity to adhere to plastic. The protocol could be implemented in quality control guidelines for scale-up cell production and cryopreservation of cells for future uses with the adding value that the methods separate cells in a label-free mode, therefore no additional manipulation occurs. The possibility to avoid antibodies-sorting method, that additionally stresses cells, are requested by the scientific community [43,44] and new methodologies are valued. We developed an innovative approach of cells’ physical characteristics measurements and their visualization in relation to the time of analysis using a scatter plot graph, which allowed a better understanding of the heterogeneity of complex biological samples. The separative potential of the NEEGA-DF methodology was already proven in expanded stem cells from amniotic fluid where we identified the most staminal subpopulation based only on cells profile [24] and dimensional characterization was manual and could not cover all cells analyzed. In this work, the cell imaging process was implemented and new information can be extrapolated, giving a general overview of the cell composition of the complex biological sample. Often geometrical features of cells are associated with differentiation potential and staminality, even though these studies involve 2D system and the use of special substrates. As an example, using high content imaging, Marklein et al. [45] demonstrated that nuclear morphological profiles of mesenchymal stromal cells had distinct morphological features that were highly predictive of MSC mineralization capabilities. In addition, time lapse imaging of 2D cell cultures in combination with morphological cell analysis are increasingly used to assess, track and even predict MSCs differentiation phenotypic outcomes. Dimension, circularity and aspect/ratio are the main geometrical characteristics that define cells. All these features can be obtained during the cell fractionation and related to functional ability after genetic and differentiation assessments. Hence, by converting the geometrical characteristics of sorted cells into a morphological fingerprint of the biological sample, this information may be used to improve cell culture, sorting of sub-population of interest and as we proved in this work as a prognostic marker to deduce vitality of primary cells.

## 5. Conclusions

In conclusion, we proved how the NEEGA-DF method and its technological implementation within Celector^®^ technology are able to predict the outcome of the isolation of AECs using the fractogram, the fingerprint of the sample, in a very short time. Moreover, the data output of the post-processing imaging adds a new type of information of the complexity of the sample to better understand its composition and cell features for its use in therapy applications.

## 6. Patents

Celector^®^ is based on a technology patented in Italy (no. IT1371772, “Method and Device to separate totipotent stem cells”), in USA, and in Canada (no. 8263359 US en. CA2649234, “Method and device to separate stem cells”).

Stem Sel^®^ has also an Italian patent (IT1426514, “Device for the Fractionation of Objects and Fractionation Method, allowed 2016).
